# Assessing the Impact of Early Nutritional Intervention on Pediatric Celiac Disease Management: A Prospective Cohort Study

**DOI:** 10.7759/cureus.76059

**Published:** 2024-12-20

**Authors:** Naveed Muhammad, Abdul Ahad, Nasar Rashid, Rabia Gul, Muhammad Arsalan Tariq, Anam Nazir

**Affiliations:** 1 Pediatrics, Khyber Teaching Hospital, Peshawar, PAK; 2 Pediatrics, Regional Hospital Mullingar, Mullingar, IRL; 3 Pediatrics, Ninewells Children's Hospital, Dundee, GBR; 4 Pediatrics, Hayatabad Medical Complex, Peshawar, PAK; 5 Pediatrics and Child Health, Hayatabad Medical Complex, Peshawar, PAK; 6 Pediatrics, Royal Hospital for Children and Young People, Edinburgh, GBR; 7 Pediatrics, Royal Aberdeen Children Hospital, Aberdeen, GBR; 8 Medicine, Basic Health Unit 155/wb, Vehari, PAK; 9 Medicine, Bahawal Victoria Hospital, Bahawalpur, PAK

**Keywords:** celiac disease, gluten-free diet, growth outcomes, nutritional intervention, pediatric

## Abstract

Background: Celiac disease is a chronic autoimmune condition requiring lifelong adherence to a gluten-free diet, particularly in children, to prevent nutritional deficiencies and developmental delays.

Objective: The objective of study was to evaluate the effects of early nutritional intervention on the management and health outcomes of children diagnosed with celiac disease.

Methodology: A prospective, longitudinal cohort study was conducted over two years (July 2019-July 2021). A customized gluten-free meal plan and any necessary dietary supplements were given to the participants. Baseline evaluations and six-monthly follow-ups were used to gather data on growth, nutritional status, and dietary compliance. SPSS was used for the statistical analysis, and a significance threshold of p<0.05 was established.

Results: Significant improvements were observed in growth metrics, with mean height increasing from 145.67 cm to 157.48 cm and weight from 36.29 kg to 46.24 kg over 24 months (p < 0.001). Nutritional status also improved, with serum iron levels rising from 45.19 µg/dL to 76.89 µg/dL and vitamin D from 20.14 ng/mL to 44.22 ng/mL (p < 0.001). Dietary adherence increased from 84.37% to 94.62%.

Conclusion: Growth and nutritional results in children with celiac disease are greatly improved with nutritional intervention.

## Introduction

A chronic autoimmune condition that affects around 1% of people worldwide, celiac disease is defined by an immune reaction to gluten consumption that destroys the small intestine and impairs nutritional absorption [1,2]. Although celiac disease may strike at any age, there are particular developmental hazards when it first manifests in childhood [3]. Children with untreated or inadequately managed celiac disease may have nutritional deficits, development delays, and an increased risk of developing other autoimmune diseases in the future [4]. Therefore, mitigating these consequences and fostering better long-term prognoses need effective early treatment [5].

Although early dietary intervention has shown promise in improving celiac disease treatment, little is known about the full extent of its influence, especially in juvenile cases [6]. Research indicates that children with celiac disease may benefit from a gluten-free diet from a young age in terms of increased immunological function, better development patterns, and fewer gastrointestinal issues [7]. A balanced gluten-free diet enhanced with vital vitamins and minerals is one kind of nutritional treatment that may be crucial in reducing the disease's harmful effects on development and growth [8]. To improve patient outcomes, further research is necessary to fully understand the precise dietary modifications, timing, and long-term advantages of early intervention [9].

Furthermore, a more thorough analysis of dietary changes during these crucial years is necessary because pediatric celiac disease often coexists with the fast developing phases of early childhood [10]. Gluten exclusion is the main focus of current treatment recommendations, but they provide little advice on portion management, ideal nutritional balance, and whether developing children need extra supplements. A organized and customized dietary plan that takes into consideration each child's particular requirements might help prevent unintentional nutritional imbalances or deficiencies that could arise from this lack of accuracy [11].

Therefore, evaluating the effects of early nutritional interventions on the management of juvenile celiac disease may help improve existing treatment modalities by providing focused dietary recommendations that promote the health of afflicted children both now and in the future.

Research objective

The objective of study was to evaluate the effects of early nutritional intervention on the management and health outcomes of children diagnosed with celiac disease.

## Materials and methods

Study design and setting

This prospective, longitudinal cohort study was conducted at Hayatabad Medical Complex and Khyber Teaching Hospital (KTH) over a two-year period, from July 2019 to July 2021.

Inclusion and exclusion criteria

Children who were newly diagnosed with celiac disease throughout the research period, ranging in age from 8-16 years, were included in the study. Intestinal biopsies and serological tests (positive anti-tissue transglutaminase antibodies) were used to confirm the diagnosis. Children were only included if they had a verified diagnosis and if their family agreed to follow the recommended intervention strategy. To guarantee accuracy in assessing the effects of early nutritional interventions on newly diagnosed cases, exclusion criteria included children who were already following a gluten-free diet before diagnosis, those who had other chronic gastrointestinal conditions (like inflammatory bowel disease), those who had congenital metabolic disorders, and those who had a history of not following medical advice.

Sample size

To calculate the sample size for the study assessing the impact of early nutritional intervention on pediatric celiac disease management, accounting for a 10% dropout rate, we used the WHO formula for proportions: n = z^2^.p.(1-p)/E^2^. Assuming a Z-value of 1.96 for a 95% confidence level, an estimated proportion p of 0.7 (indicating a 70% improvement), and a margin of error E of 0.05, the calculation yields approximately n = (1.96)^2^x0.7x0.3/(0.05)^2^ ​≈ 323 participants. To account for the anticipated 10% dropout rate, we adjust the sample size using the formula n adjusted = n/(1−dropout rate) = 323/0.9​, resulting in a final target sample size of 360 participants to ensure sufficient power for the study.

Data collection

Follow-ups were carried out every six months to track nutritional status, growth, and dietary compliance after baseline data collection. To create nutritional and health baselines, baseline evaluations included a thorough food history, anthropometric measures (height, weight, and BMI), and laboratory tests (serum iron, calcium, vitamin D, and complete blood count). Under the guidance of a dietician, the intervention included a customized gluten-free food plan along with suggested supplements (iron, calcium, and vitamin D) based on individual deficits. Each follow-up visit included food memory tests, a reassessment of lab measurements, and an evaluation of the participants' growth metrics and gluten-free diet compliance.

Nutritional intervention protocol

A customized, well-balanced gluten-free diet plan tailored to each participant's age-specific calorie and nutritional requirements was provided. Dietitians gave information and advice on how to prepare gluten-free meals, where to get foods, and how to read product labels. To promote adherence and handle nutritional issues, a nutritionist called families once a month. Depending on the results of the first and follow-up evaluations, additional supplements, such as multivitamins and minerals, were recommended as needed.

Statistical analysis

We used SPSS software, version 26, to analyze the data. Baseline characteristics and follow-up results were summarized using descriptive statistics (means, standard deviations, frequencies). Growth metrics, laboratory data, and dietary adherence were compared over time using paired t-tests, with a significance threshold of p<0.05.

Ethics approval

The institutional review board (IRB) gave the research ethical clearance (approval no. 199/OD/HMC/2019). All participants' parents or guardians provided written informed permission after being made aware of the study's goals, methods, and any hazards. All patient data was anonymized and securely retained for use in this research solely in order to protect privacy.

## Results

The baseline demographics and nutritional parameters of pediatric celiac disease patients are shown in Table 1. The patients' mean age is 12.5 years (± 2.5), and they are evenly divided between 180 boys (50.00%) and 180 girls (50.00%). Bread (320 patients, 88.89%), pasta (280 patients, 77.78%), cereals (250 patients, 69.44%), and processed foods (200 patients, 55.56%) were the most common dietary sources of gluten. The average height, weight, and BMI were 145.67 cm (± 10.35), 36.29 kg (± 8.48), and 17.23 kg/m² (± 2.29), according to anthropometric measures.

**Table 1:** Baseline demographics and nutritional characteristics of pediatric patients with celiac disease

Characteristic		Number of Patients (n, %)
Age (years)	Mean ± SD	12.5 ± 2.5
Gender	Male	180 (50.00%)
	Female	180 (50.00%)
Typical Dietary Sources of Gluten	Bread	320 (88.89%)
	Pasta	280 (77.78%)
	Cereals	250 (69.44%)
	Processed Foods	200 (55.56%)
Anthropometric Measurements	Height (cm)	145.67 ± 10.35
	Weight (kg)	36.29 ± 8.48
	Body Mass Index (kg/m²)	17.23 ± 2.29

Pediatric patients with celiac disease had mean serum iron levels of 45.19 µg/dL (± 15.42), mean serum calcium levels of 8.95 mg/dL (± 1.05), and mean serum vitamin D levels of 20.14 ng/mL (± 5.58) according to Table 2, which summarizes the preliminary laboratory evaluations of nutritional and hematological parameters. Furthermore, the average white blood cell count was 7,891 cells/µL (± 234), the average platelet count was 246,000 cells/µL (± 12,000), and the average hemoglobin concentration was 13.34 g/dL (± 1.23).

**Table 2:** Initial laboratory assessments of nutritional and hematological parameters in pediatric celiac disease

Laboratory Parameter	Mean (± SD)
Serum Iron (µg/dL)	45.19 ± 15.42
Serum Calcium (mg/dL)	8.95 ± 1.05
Serum Vitamin D (ng/mL)	20.14 ± 5.58
Hemoglobin (g/dL)	13.34 ± 1.23
White Blood Cell Count (WBC/µL)	7,891 ± 234
Platelet Count (×10³/µL)	246,000 ± 12,000

The trends of nutritional supplements and dietary adherence in juvenile celiac disease patients throughout time are shown in Table 3. By six months, 84.37% (± 9.82%) of patients were following the gluten-free diet, and 61.21% (± 4.53%) of patients were taking nutritional supplements, which included an average of 20 mg of iron, 500 mg of calcium, and 1,000 IU of vitamin D. With doses of 25 mg of iron, 600 mg of calcium, and 1500 IU of vitamin D, the proportion of participants getting supplements climbed to 73.45% (± 6.32%) and adherence to 89.78% (± 8.14%) at the 12-month mark. By 18 months, 91.56% (± 5.67%) of participants were taking supplements, with 79.84% (± 5.45%) taking higher amounts of 2000 IU of vitamin D, 700 mg of calcium, and 30 mg of iron. Ultimately, at 24 months, 94.62% (± 3.78%) of the participants were following the gluten-free diet, and 84.90% (± 4.21%) were taking supplements. The amount of iron was maintained at 30 mg, but calcium and vitamin D were increased to 800 mg and 2500 IU, respectively.

**Table 3:** Dietary adherence and nutritional supplementation patterns over time in pediatric patients with celiac disease The values in parentheses represent the SD of the percentages.

Time Point	Adherence to Gluten-Free Diet	Receiving Nutritional Supplements	Mean Supplementation Dosage (if applicable)
6 Months	84.37% (± 9.82%)	61.21% (± 4.53%)	Iron: 20 mg, Calcium: 500 mg, Vitamin D: 1,000 IU
12 Months	89.78% (± 8.14%)	73.45% (± 6.32%)	Iron: 25 mg, Calcium: 600 mg, Vitamin D: 1,500 IU
18 Months	91.56% (± 5.67%)	79.84% (± 5.45%)	Iron: 30 mg, Calcium: 700 mg, Vitamin D: 2,000 IU
24 Months	94.62% (± 3.78%)	84.90% (± 4.21%)	Iron: 30 mg, Calcium: 800 mg, Vitamin D: 2,500 IU

The 24-month longitudinal changes in development and nutritional status among juvenile celiac disease patients are shown in Table 4. Numerous metrics show notable improvements, according to the data. With a p-value of less than 0.001, the mean height rose from 145.67 cm (± 10.35) at baseline to 157.48 cm (± 7.54) at 24 months. Additionally, weight increased significantly during the same time period, going from 36.29 kg (± 8.48) to 46.24 kg (± 5.79; p < 0.001). With a p-value of less than 0.01 the BMI rose from 17.23 kg/m² (± 2.29) to 19.36 kg/m² (± 1.65). Serum calcium rose from 8.95 mg/dL (± 1.05) to 11.27 mg/dL (± 0.41) (p < 0.001), while serum iron levels improved dramatically from 45.19 µg/dL (± 15.42) to 76.89 µg/dL (± 9.42) (p < 0.001). Additionally, serum vitamin D levels significantly improved from 20.14 ng/mL (± 5.58) to 44.22 ng/mL (± 4.32), with a p-value of <0.001, suggesting that these patients' nutritional condition had improved overall.

**Table 4:** Longitudinal changes in growth and nutritional status in pediatric patients with celiac disease The p-values were calculated using repeated measures ANOVA to assess changes over time. ANOVA: Analysis of variance

Parameter	Baseline	6 Months	12 Months	18 Months	24 Months	p-value
Height (cm)	145.67 ± 10.35	148.42 ± 9.78	151.12 ± 8.81	154.23 ± 8.17	157.48 ± 7.54	<0.001
Weight (kg)	36.29 ± 8.48	38.56 ± 7.36	41.05 ± 6.90	43.68 ± 6.51	46.24 ± 5.79	<0.001
Body Mass Index (kg/m²)	17.23 ± 2.29	17.65 ± 2.14	18.06 ± 1.88	18.49 ± 1.91	19.36 ± 1.65	<0.01
Serum Iron (µg/dL)	45.19 ± 15.42	53.85 ± 12.18	61.28 ± 11.12	69.25 ± 10.58	76.89 ± 9.42	<0.001
Serum Calcium (mg/dL)	8.95 ± 1.05	9.49 ± 0.76	10.12 ± 0.64	10.78 ± 0.52	11.27 ± 0.41	<0.001
Serum Vitamin D (ng/mL)	20.14 ± 5.58	25.43 ± 6.25	31.34 ± 5.48	37.19 ± 5.09	44.22 ± 4.32	<0.001

## Discussion

A gluten-free diet must be strictly followed by people with celiac disease, a chronic autoimmune condition, especially in young children, to avoid developmental retardation and nutritional deficits. Significant improvements in a number of health indices were found in this research that assessed the impact of early dietary intervention on juvenile celiac disease patients. Our cohort's mean height and weight at baseline were 145.67 cm (± 10.35) and 36.29 kg (± 8.48), respectively. With mean height reaching 157.48 cm (± 7.54) and weight reaching 46.24 kg (± 5.79) over the 24-month follow-up, these measurements increased significantly, indicating a positive growth trajectory. When compared to age-appropriate standards, these increases in height and weight were consistent with the expected growth for the participants' age groups, as outlined by the World Health Organization and the Centers for Disease Control and Prevention. These findings align with earlier studies that demonstrated similar improvements in growth metrics among children following a gluten-free diet from an early age [12,13].

Additionally, our investigation showed that laboratory parameters had significantly improved. By the end of 24 months, the mean serum iron level had significantly improved, rising from 45.19 µg/dL (± 15.42) at baseline to 76.89 µg/dL (± 9.42). These results are consistent with other studies that showed early dietary intervention enhanced iron levels in children, lowering the risk of iron deficiency anemia, which is often seen in this group [14]. Additionally, by the conclusion of the research, blood calcium and vitamin D levels increased from their baseline values of 8.95 mg/dL (± 1.05) and 20.14 ng/mL (± 5.58) to 11.27 mg/dL (± 0.41) and 44.22 ng/mL (± 4.32). This rise supports earlier research that highlighted the effect of dietary interventions in raising calcium and vitamin D levels in children with celiac disease and highlights the need for customized nutritional supplements as part of celiac disease care [15].

Additionally, the percentage of those who followed the gluten-free diet increased significantly from 84.37% (± 9.82%) at 6 months to 94.62% (± 3.78%) after 24 months. The findings of a prior longitudinal trial, which found that organized nutritional counseling considerably improved adherence among pediatric patients, are in line with this slow increase in adherence [16]. This adherence was probably influenced by the focus on regular check-ups and customized meal programs, which included nutritional supplements. This highlights the need for ongoing assistance in properly treating celiac disease.

Study strengths and limitations

This research has a number of advantages, such as a prospective, longitudinal design and a large enough sample size to improve the results' dependability. A thorough analysis of growth indicators, nutritional status, and gluten-free diet compliance offers a reliable assessment of the effects of early nutritional intervention in children with celiac disease. Nevertheless, there are several significant drawbacks. The single-center design of the research and the particular participant demography may limit its generalizability, which may impact how broadly the findings may be applied. Furthermore, while the study demonstrates significant improvements in the anthropometric and nutritional parameters, we acknowledge that the lack of a control or comparison group could limit the ability to directly assess these changes relative to a healthy population. The study was specifically designed to evaluate the effects of early nutritional intervention in newly diagnosed pediatric celiac disease patients. Future research could benefit from including a matched control group to provide further context to these findings and better compare the impact of the intervention.

## Conclusions

This research emphasizes how important early dietary intervention is for managing celiac disease in children. Over a 24-month period, the results show significant increases in participants' development, nutritional status, and adherence to a gluten-free diet. Children's growth metrics, serum iron, calcium, and vitamin D levels, and dietary adherence all improved when a customized gluten-free diet enriched with vital vitamins and minerals was put into place. These findings support the need for further study to improve dietary recommendations and intervention procedures by highlighting the importance of early and organized nutritional interventions in maximizing health outcomes for children with celiac disease.
